# Development of childhood asthma prediction models using machine learning approaches

**DOI:** 10.1002/clt2.12076

**Published:** 2021-11-07

**Authors:** Dilini M. Kothalawala, Clare S. Murray, Angela Simpson, Adnan Custovic, William J. Tapper, S. Hasan Arshad, John W. Holloway, Faisal I. Rezwan, STELAR/UNICORN investigators

**Affiliations:** ^1^ Human Development and Health Faculty of Medicine University of Southampton Southampton UK; ^2^ NIHR Southampton Biomedical Research Centre University Hospital Southampton Southampton UK; ^3^ Division of Infection, Immunity, and Respiratory Medicine School of Biological Sciences University of Manchester Manchester University Hospital NHS Foundation Trust Manchester Academic Health Science Centre Manchester UK; ^4^ National Heart and Lung Institute Imperial College of Science, Technology, and Medicine London UK; ^5^ The David Hide Asthma and Allergy Research Centre St. Mary's Hospital Isle of Wight UK; ^6^ Clinical and Experimental Sciences Faculty of Medicine University of Southampton Southampton UK; ^7^ Department of Computer Science Aberystwyth University Aberystwyth UK

**Keywords:** asthma, childhood, machine learning, prediction, Asthma, Kindheit, maschinelles Lernen, Prognose

## Abstract

**Background:**

Respiratory symptoms are common in early life and often transient. It is difficult to identify in which children these will persist and result in asthma. Machine learning (ML) approaches have the potential for better predictive performance and generalisability over existing childhood asthma prediction models. This study applied ML approaches to predict school‐age asthma (age 10) in early life (Childhood Asthma Prediction in Early life, CAPE model) and at preschool age (Childhood Asthma Prediction at Preschool age, CAPP model).

**Methods:**

Clinical and environmental exposure data was collected from children enrolled in the Isle of Wight Birth Cohort (*N* = 1368, ∼15% asthma prevalence). Recursive Feature Elimination (RFE) identified an optimal subset of features predictive of school‐age asthma for each model. Seven state‐of‐the‐art ML classification algorithms were used to develop prognostic models. Training was performed by applying fivefold cross‐validation, imputation, and resampling. Predictive performance was evaluated on the test set. Models were further externally validated in the Manchester Asthma and Allergy Study (MAAS) cohort.

**Results:**

RFE identified eight and twelve predictors for the CAPE and CAPP models, respectively. Support Vector Machine (SVM) algorithms provided the best performance for both the CAPE (area under the receiver operating characteristic curve, AUC = 0.71) and CAPP (AUC = 0.82) models. Both models demonstrated good generalisability in MAAS (CAPE 8‐year = 0.71, 11‐year = 0.71, CAPP 8‐year = 0.83, 11‐year = 0.79) and excellent sensitivity to predict a subgroup of persistent wheezers.

**Conclusion:**

Using ML approaches improved upon the predictive performance of existing regression‐based models, with good generalisability and ability to rule in asthma and predict persistent wheeze.

## 1 INTRODUCTION

Childhood asthma is highly heterogeneous, with numerous factors contributing towards its development, persistence and severity.^1^, ^2^, ^3^ Despite approximately 80% of asthmatic children developing symptoms (such as wheeze) before the age of six, these clinical symptoms are neither universally present in early life among all future asthmatics nor specific to asthma.^4^ With the added difficulty of making an objective asthma diagnosis before the age of five, both under‐treatment and over‐treatment of wheezing disorders are common in early life.^5^, ^6^

The ability to predict the development of school‐age asthma can help to identify high‐risk preschool children and distinguish them from children whose symptoms are likely to be transient.^7^ Furthermore, early prediction of asthma susceptibility will be critical for the successful implementation of potential primary prevention strategies to reduce the risk of developing asthma.

A recent systematic review identified twenty‐one logistic regression‐based models for predicting childhood asthma.^8^ However, none of these models have been implemented into standard clinical practice, possibly due to relatively weak predictive power, poor generalisability and need for specialised clinical testing. The review further proposed that regression‐based methods for predicting childhood asthma may have been exhausted, with the identified models offering similar predictive power to each other and being unable to be significantly improved upon.^8^

Machine learning approaches have increasingly been applied to a wide range of healthcare problems due to their ability to integrate large quantities of heterogeneous data, handle complex interactions between variables and identify patterns within data.^9^ Particularly for disease prediction, where interactions between biological variables are complex, machine learning approaches have the potential to identify novel predictors which may have been previously overlooked by regression‐based approaches.^9^, ^10^, ^11^ Furthermore, application of methods to reduce model overfitting may address the poor generalisability of existing prediction models in independent populations. Machine learning approaches have shown promise in predicting a variety of clinical asthma outcomes, phenotypes and decisions,^12^, ^13^, ^14^, ^15^, ^16^ including the diagnostic or prognostic prediction of school‐age asthma development.^17^, ^18^, ^19^, ^20^, ^21^, ^22^, ^23^, ^24^, ^25^ While these studies tend to offer improved predictive performance, none of these studies support their findings with external validations of their models or explain how their “black‐box” models (where relevant) arrive at their predictions. Without these two components, machine learning models will fail to obtain the trust of physicians and continue to be limited in their clinical utility, regardless of the superior prediction accuracy they may offer.^26^, ^27^

This study aimed to utilise machine learning approaches to improve upon the performance of traditional regression methods and develop explainable and independently validated prediction models for childhood asthma. Two prognostic prediction models, the Childhood Asthma Prediction in Early‐life (CAPE) and Childhood Asthma Prediction at Preschool‐age (CAPP) models, were developed to predict school‐age asthma at 10 years, within a general population‐based cohort, using information available from the first two years and first four years of life, respectively.

## 2 METHODS

### 2.1 Developmental study population

Data was obtained from 1456 individuals from the Isle of Wight Birth Cohort (IOWBC). Study recruitment and participant details have been previously described^28^ (see supporting information S1). Ethical approval was obtained from the Isle of Wight Local Research Ethics Committee at recruitment and 1‐, 2‐ and 4‐year assessments (No. 05/89) and 10‐year assessments including genetic studies (No. 18/98). Prior to participation in the study at each follow‐up, written informed consent was obtained from parents of children, and assent from children (where applicable). This study received approval from the University of Southampton Faculty of Medicine ethics committee (ERGO number 46033.R1).

### 2.2 Prediction outcome

School‐age asthma, evaluated at age 10, was defined as “a doctor diagnosis of asthma ever and at least one episode of wheezing or use of asthma medication in the last 12 months”. Only individuals with a reported asthma status at the 10‐year follow‐up were included in the analyses (*n* = 1368).

### 2.3 Candidate predictors

Fifty‐four candidate predictors previously reported to be associated with childhood asthma, and for which data was available in the IOWBC, were identified (Table E1). Candidate predictors included data on subject demographics, lifestyle, clinical symptoms of allergy and asthma and environmental exposures collected across three time points: at birth (prenatal and perinatal data), early life (combined exposure at either the 1‐year or the 2‐year follow‐ups) and at preschool age (4‐year follow‐up).

### 2.4 Model development

**FIGURE 1:**
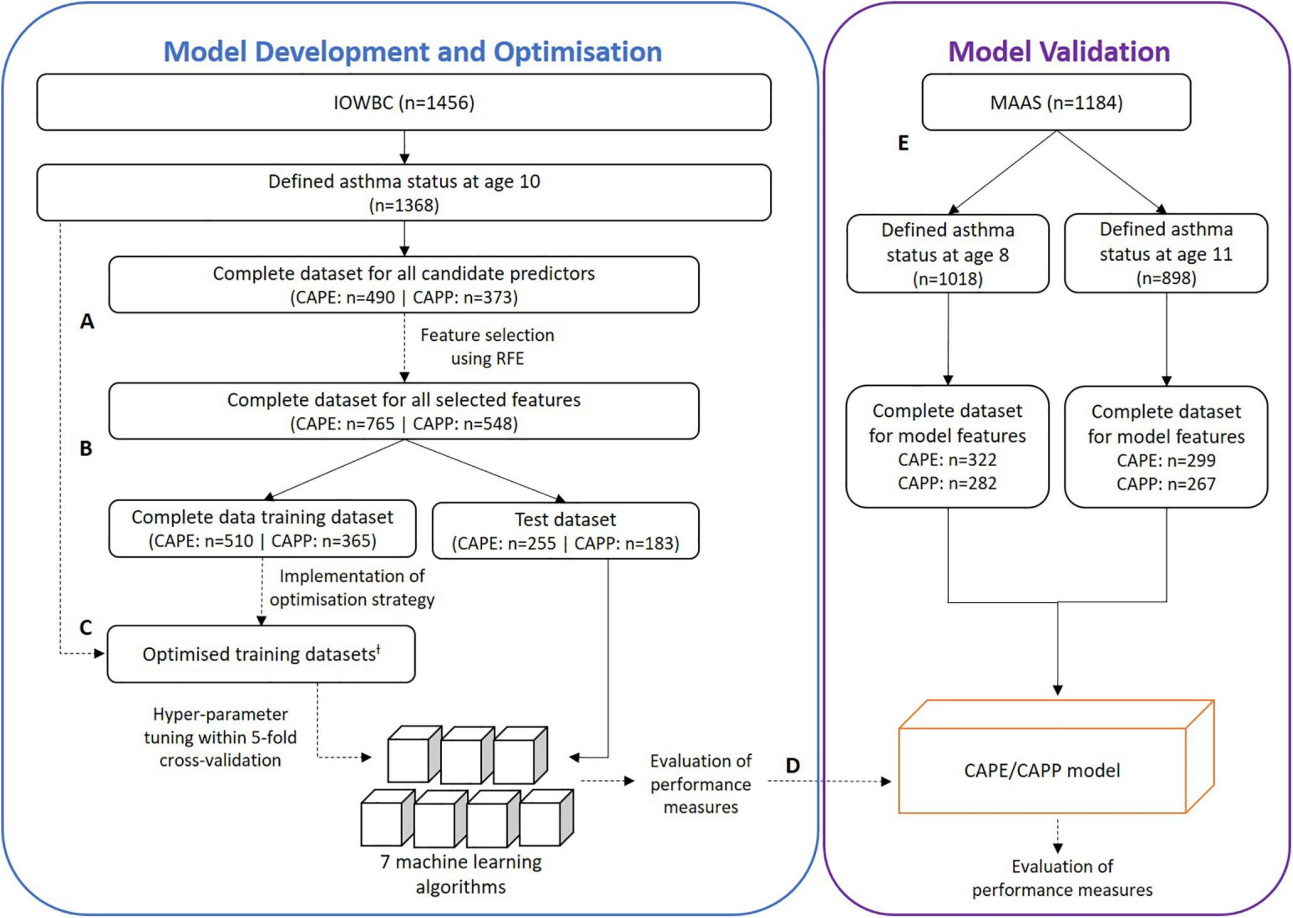
Workflow for the development and validation of asthma prediction models using machine learning approaches. Model development in the Isle of Wight Birth Cohort (IOWBC) was performed independently in for the construction of the CAPE and CAPP tools. (A) Feature selection was performed using only individuals with complete data for all candidate predictors. (B, C) Seven machine learning classifiers (two support vector machines with different kernel functions (linear and radial basis function), naïve Bayes classifier, decision tree, multilayer perceptron, random forest and K‐nearest neighbours) were developed. Models were developed using complete data for the subset of features identified from feature selection (B), and subsequently redeveloped using optimised training datasets (C). Training dataset optimisation consisted of the step‐wise application of imputation and resampling (oversampling using ADASYN and random undersampling) to the entire IOWBC dataset not allocated to the test dataset, including those with missing predictor data (CAPE: *n* = 1113; CAPP: *n* = 1185). (D) The best CAPE and CAPP models were selected based on performance in the test set. (E) Selected models were externally validated to predict school‐age asthma at ages 8 and 11 years in an independent population (Manchester Asthma and Allergy Study, MAAS). ^†^The performance of the best CAPE model was developed on the complete training dataset, undersampled to balance class proportions (*n* = 136). The best CAPP model was developed on the complete training dataset, with cases oversampled by 300% and controls undersampled to balance class proportions (*n* = 408)

All stages of model development were performed independently for the CAPE and CAPP models (Figure 1).

### 2.5 Feature selection

For each model, feature selection was performed on the complete dataset for all available candidate predictors (without any missing values) using Recursive Feature Elimination (RFE) with a random forest algorithm, using fivefold cross‐validation (see supporting information S1).

### 2.6 Model construction and optimisation

To identify the best classification algorithm, seven machine learning classifiers were implemented: two support vector machines (SVM) (linear and radial basis (RBF) kernel functions), decision tree, random forest, naive Bayes, multilayer perceptron, and K‐Nearest Neighbours (see supporting information S1).

Each machine learning algorithm was initially trained and evaluated on the subset of individuals who had complete data for the predictors selected through RFE. The dataset was split (ratio of 2:1, preserving class proportions) into a training and holdout test set for model development and validation, respectively (Figure 1). Within a fivefold cross‐validation, the hyperparameters for each model were tuned using a grid search, optimising for its balanced accuracy (see supporting information S1, Table E2).

The training dataset was then optimised to further improve the performance of the classification algorithms. Multiple imputation using Multivariate Imputation by Chain Equations (MICE),^29^ oversampling using an adaptive synthetic sampling approach (ADASYN),^30^ and random under‐sampling were implemented in a stepwise approach to address the degree of missing data and class imbalance in the training set (see supporting information S1). The seven algorithms were then redeveloped, with hyperparameters retuned, on each optimised training set to identify the best asthma prediction model(s) and tested on the same holdout test set (Figure 1).

The best CAPE and CAPP models were selected based on their discriminative performance on the test set using the area under the receiver operating characteristics curve (AUC). Sensitivity, specificity, positive and negative predictive values (PPV and NPV), positive and negative likelihood ratios (LR+ and LR−), balanced accuracy, F1‐score and Brier score were reported at the optimal threshold that maximized the Youden's Index, with 2000 bootstrap samples used to calculate 95% confidence intervals for the performance measures.

### 2.7 External validation

The best performing models were validated in the Manchester Asthma and Allergy Study (MAAS) cohort^31^ to predict school‐age asthma at ages eight and eleven (Figure 1, see supporting information S1). Data extracted from MAAS was closely matched to maximise the similarity of predictor and outcome definitions used in the development cohort (Table E3).

### 2.8 Sensitivity analyses

Sensitivity analyses were conducted to comprehensively evaluate the developed models, including evaluations of (i) their generalisability in high risk subgroups; (ii) their robustness to predict an alternative definition of school‐age asthma; (iii) the resolution of the predictions to distinguish between individuals presenting with distinct wheeze phenotypes throughout childhood and adolescence; and (iv) their performance compared to similar regression‐based models (see supporting information S1).

### 2.9 Explaining the ‘black‐box’ models

SHapley Additive exPlanations (SHAP)^32^ were used to evaluate feature importance and provide global explanations for how predictions were made by the CAPE and CAPP models (see supporting information S1). Examples of how SHAP can be used locally to explain individual predictions were also provided.

## 3 RESULTS

In the IOWBC, 1368 enrolled participants had a defined asthma outcome at age 10, of whom 201 (14.69%) were asthmatic. Baseline characteristics between individuals with complete data were largely comparable with the full IOWBC dataset (Table E4).

### 3.1 Childhood Asthma Prediction in Early‐life (CAPE) model

**FIGURE 2:**
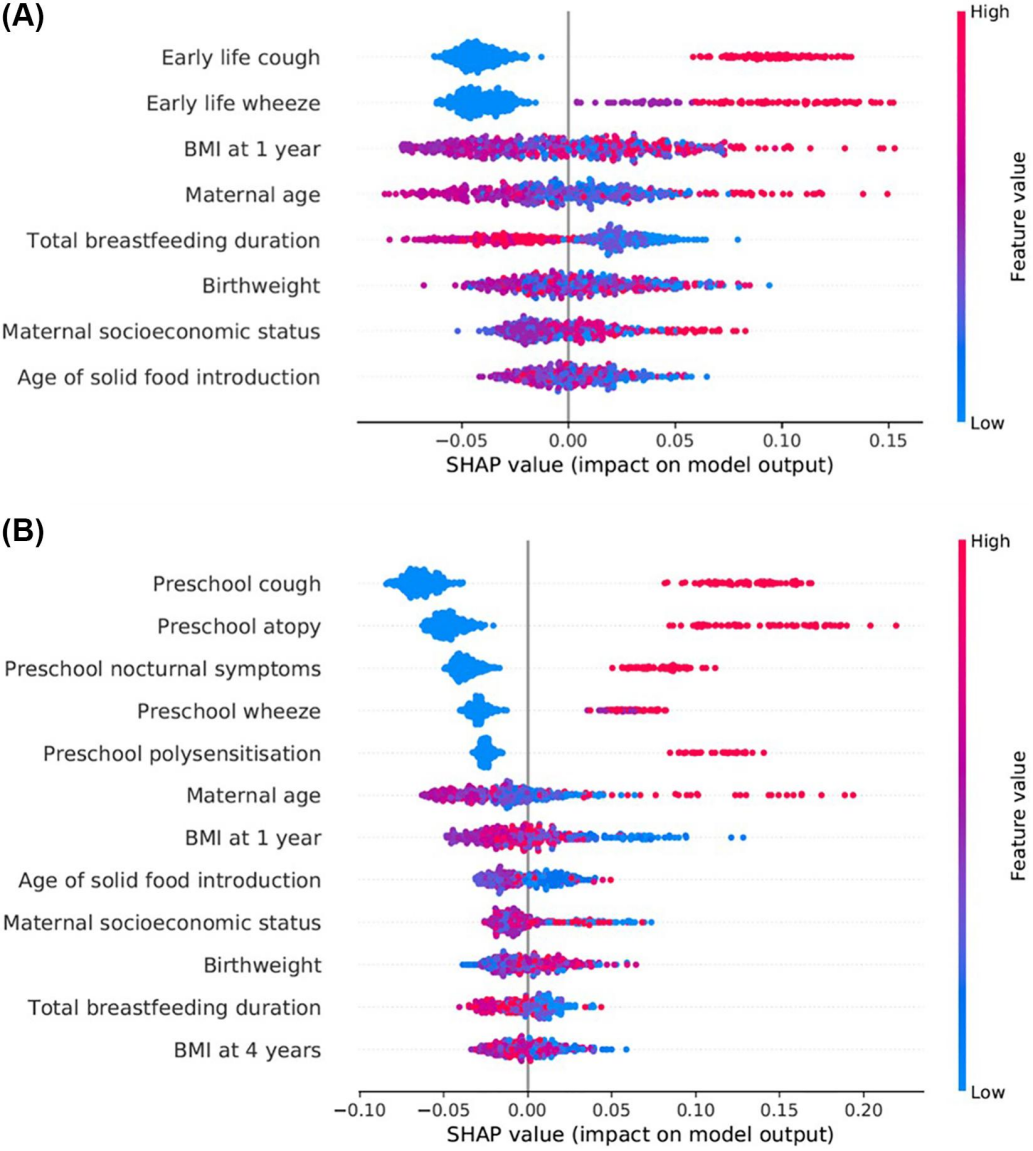
SHAP feature explanations for the CAPE and CAPP models. The SHAP summary plots describe the contribution of the features selected during RFE for inclusion in the CAPE (A) and CAPP models (B). Predictors are listed in descending order of their SHAP value. The higher the SHAP value, the larger its contribution on model predictions. Each dot in each predictor row corresponds to a separate individual. The placing of the dot along the *x*‐axis represents the contribution of the predictor in the individual's asthma prediction. The colour of the dot refers to the feature value, with higher values in red and lower values in blue. For example, early life cough offers the highest contribution to the random forest model, with higher values (presence of early life cough) having a positive contribution towards a prediction of asthma. The absence of early life cough (blue dots) reduces the impact/contribution of the model delivering a prediction of asthma

Complete data on all 39 predictors collected by age two was available for 490 individuals. RFE identified a subset of eight predictors for inclusion in the CAPE model, with an average balanced accuracy of 64.49%. Figure 2A details the feature importance, direction, and magnitude of asthma risk for each selected predictor based on SHAP. Complete data for these eight predictors was available for 765 individuals; 510 (68 asthmatics) and 255 (34 asthmatics) individuals were allocated to the initial training and test sets, respectively. An SVM classifier (RBF kernel) was the best performing classification algorithm for the CAPE model (AUC = 0.71, Brier score = 0.21) (Table 1A).

**TABLE 1:** Performance of the CAPE and CAPP models

A. Performance of the CAPE model											
	Dataset	Sample size (no. asthmatic)	Balanced accuracy	AUC	Sensitivity	Specificity	PPV	NPV	LR+	LR‐	F1 score
Development—IOWBC: 10 years	Training^a^	136 (68 asthmatic)	0.65 (0.57, 0.74)	0.76 (0.68, 0.84)	0.56 (0.44, 0.68)	0.75 (0.63, 0.85)	0.69 (0.59, 0.79)	0.63 (0.56, 0.71)	2.24 (1.44, 3.80)	0.59 (0.42, 0.78)	0.62 (0.52, 0.71)
	Testing	255 (34 asthmatic)	0.71 (0.62, 0.78)	0.71 (0.61, 0.80)	0.74 (0.56, 0.88)	0.68 (0.62, 0.74)	0.26 (0.21, 0.32)	0.94 (0.91, 0.97)	2.29 (1.69, 3.01)	0.39 (0.18, 0.63)	0.38 (0.31, 0.46)
External Validation—MAAS: 8 years	Unselected	322 (38 asthmatic)	0.67 (0.60, 0.74)	0.71 (0.63, 0.79)	0.84 (0.71, 0.95)	0.51 (0.45, 0.56)	0.19 (0.16, 0.21)	0.96 (0.93, 0.99)	1.71 (1.40, 2.03)	0.31 (0.10, 0.57)	0.30 (0.26, 0.35)
	High‐risk^c^	208 (31 asthmatic)	0.66 (0.59, 0.73)	0.71 (0.61, 0.80)	0.87 (0.74, 0.97)	0.46 (0.39, 0.53)	0.22 (0.19, 0.25)	0.95 (0.91, 0.99)	1.61 (1.31, 1.95)	0.28 (0.06, 0.59)	0.35 (0.30, 0.40)
	High‐risk^d^	81 (16 asthmatic)	0.57 (0.45, 0.67)	0.64 (0.47, 0.80)	0.81 (0.63, 1.00)	0.32 (0.22, 0.43)	0.23 (0.18, 0.28)	0.88 (0.75, 1.00)	1.20 (0.86, 1.56)	0.58 (0.00, 1.35)	0.36 (0.27, 0.43)
External Validation—MAAS: 11 years	Unselected	299 (32 asthmatic)	0.68 (0.60, 0.74)	0.71 (0.62, 0.79)	0.84 (0.72, 0.97)	0.51 (0.45, 0.57)	0.17 (0.14, 0.20)	0.96 (0.94, 0.99)	1.72 (1.39, 2.05)	0.31 (0.07, 0.58)	0.28 (0.24, 0.33)
	High‐risk^c^	192 (25 asthmatic)	0.67 (0.59, 0.74)	0.71 (0.62, 0.80)	0.88 (0.76, 1.00)	0.47 (0.40, 0.54)	0.20 (0.17, 0.23)	0.96 (0.92, 1.00)	1.65 (1.34, 2.03)	0.26 (0.00, 0.57)	0.32 (0.27, 0.37)
	High‐risk^d^	72 (12 asthmatic)	0.58 (0.44, 0.69)	0.60 (0.43, 0.76)	0.83 (0.58, 1.00)	0.33 (0.22, 0.45)	0.20 (0.15, 0.25)	0.91 (0.78, 1.00)	1.25 (0.85, 1.66)	0.50 (0.00, 1.39)	0.32 (0.23, 0.40)

B. Performance of the CAPP model											
	Population	Sample size (no. asthmatic)	Balanced accuracy	AUC	Sensitivity	Specificity	PPV	NPV	LR+	LR‐	F1 score
Development—IOWBC: 10 years	Training^b^	408 (204 asthmatic)	0.78 (0.75, 0.82)	0.85 (0.81, 0.89)	0.80 (0.74, 0.82)	0.77 (0.71, 0.85)	0.78 (0.73, 0.82)	0.79 (0.75, 0.84)	3.47 (2.73, 4.61)	0.26 (0.19, 0.34)	0.79 (0.75, 0.83)
	Testing	183 (25 asthmatic)	0.80 (0.70, 0.89)	0.82 (0.71, 0.91)	0.72 (0.52, 0.88)	0.88 (0.83, 0.92)	0.47 (0.38, 0.62)	0.95 (0.92, 0.98)	5.99 (3.79, 10.11)	0.32 (0.13, 0.54)	0.56 (0.45, 0.70)
External Validation—MAAS: 8 years	Unselected	282 (33 asthmatic)	0.73 (0.64, 0.81)	0.83 (0.75, 0.90)	0.55 (0.36, 0.70)	0.91 (0.88, 0.95)	0.45 (0.33, 0.59)	0.94 (0.92, 0.96)	6.17 (3.64, 10.69)	0.50 (0.33, 0.69)	0.49 (0.36, 0.62)
	High‐risk^c^	178 (26 asthmatic)	0.70 (0.60, 0.80)	0.80 (0.70, 0.88)	0.50 (0.31, 0.69)	0.90 (0.85, 0.95)	0.46 (0.32, 0.63)	0.91 (0.89, 0.94)	5.07 (2.77, 9.95)	0.55 (0.34, 0.77)	0.48 (0.32, 0.63)
	High‐risk^d^	70 (13 asthmatic)	0.73 (0.59, 0.87)	0.80 (0.62, 0.94)	0.54 (0.23, 0.77)	0.93 (0.86, 0.98)	0.64 (0.40, 0.90)	0.90 (0.84, 0.95)	7.67 (2.92, 39.46)	0.50 (0.24, 0.81)	0.58 (0.32, 0.78)
External Validation—MAAS: 11 years	Unselected	267 (29 asthmatic)	0.73 (0.63, 0.82)	0.79 (0.68, 0.88)	0.55 (0.38, 0.72)	0.90 (0.87, 0.94)	0.41 (0.29, 0.55)	0.94 (0.92, 0.96)	5.71 (3.44, 9.85)	0.50 (0.30, 0.71)	0.47 (0.33, 0.62)
	High‐risk^c^	169 (22 asthmatic)	0.72 (0.61, 0.82)	0.76 (0.61, 0.88)	0.55 (0.36, 0.73)	0.89 (0.84, 0.94)	0.43 (0.29, 0.59)	0.93 (0.90, 0.96)	5.01 (2.75, 9.65)	0.51 (0.30, 0.74)	0.48 (0.32, 0.63)
	High‐risk^d^	64 (10 asthmatic)	0.75 (0.59, 0.90)	0.73 (0.47, 0.94)	0.60 (0.30, 0.90)	0.91 (0.83, 0.98)	0.55 (0.31, 0.86)	0.92 (0.87, 0.98)	6.48 (2.40, 32.40)	0.44 (0.11, 0.80)	0.57 (0.30, 0.78)

*Note*: Performance measures are reported, with 95% confidence intervals from 2000 bootstrap samples in brackets. Performance measures in the IOWBC test set and MAAS are evaluated at thresholds of 0.42 (CAPE model) and 0.73 (CAPP model).

^a^ The CAPE model was developed using an SVM classification algorithm using a radial basis function kernel (*C* = 45.1, gamma = 0.0054). The model was trained on the training dataset consisting of individuals with complete data, with controls under‐sampled to obtain a 1:1 class ratio.

^b^ The CAPP model was developed using an SVM classification algorithm using a linear kernel (*C* = 0.33), and was trained on the training dataset consisting of individuals with complete data, with cases oversampled by 300% and controls under‐sampled to obtain a 1:1 class ratio.

^c^ High‐risk, defined as a child having at least one parent with allergic disease (asthma, eczema or allergic rhinitis).

^d^ High‐risk, defined as a child with both parents with allergic disease (asthma, eczema or allergic rhinitis).

### 3.2 External validation of the CAPE model

**FIGURE 3:**
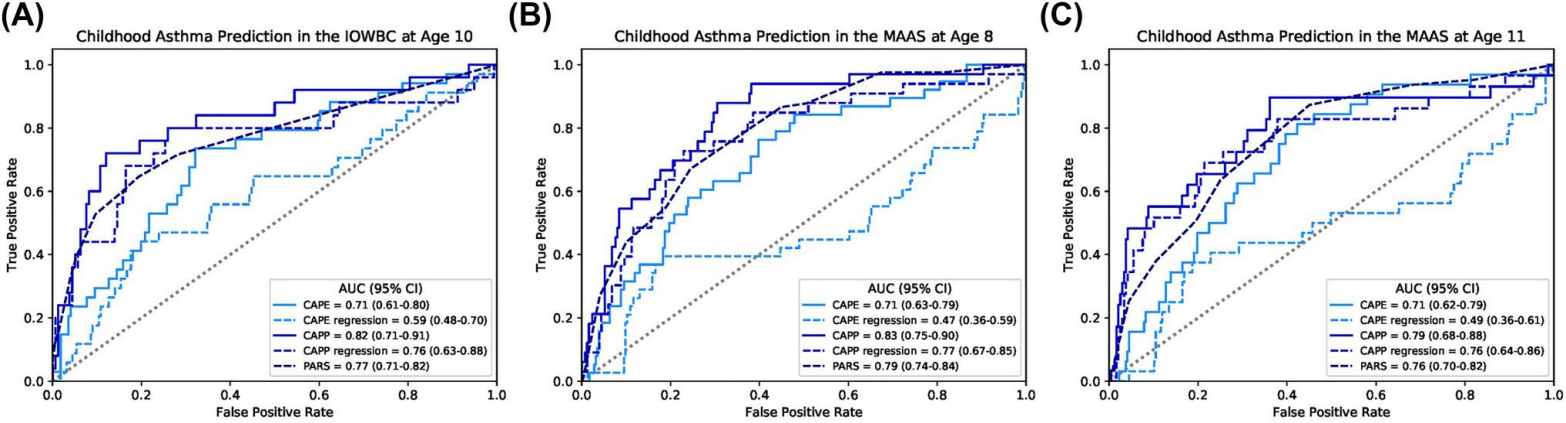
Discriminative performance of the CAPE and CAPP machine learning models. The ROC curves compare the discriminative performance of the CAPE and CAPP machine learning models, their equivalent logistic regression models and the PARS model in the IOWBC at age 10 (A) and upon validation in MAAS at age 8 years (B) and 11 years (C)

To predict the development of asthma at the 8‐year and 11‐year time‐points in MAAS, complete data on the eight CAPE predictors was available for 322 and 299 individuals, respectively. Table E5 compares the distribution of predictors in the IOWBC and MAAS. The CAPE model demonstrated moderate generalisability, maintaining an AUC = 0.71 at both 8 and 11 years (Table 1A; Figure 3), despite slight reductions in PPV. In the high‐risk subgroups, despite a 3%–4% increase in PPV, overall predictive performance decreased (Table 1A).

### 3.3 Childhood Asthma Prediction at Preschool‐age (CAPP) model

For the CAPP model, 373 individuals had complete data for all 54 candidate predictors available by age four. RFE identified an optimal subset of 12 predictors for inclusion in the model, with an average balanced accuracy of 74.93% (Figure 2B). Complete data for these 12 predictors was available for 548 individuals, of whom 365 (51 asthmatics) and 183 (25 asthmatics) individuals were assigned to the initial training and test sets, respectively. The best performing classification algorithm for the CAPP model was an SVM (linear kernel) classifier (AUC = 0.82, Brier score = 0.18) (Table 1B).

### 3.4 External validation of the CAPP model

For validation of the CAPP model in MAAS at the 8‐year and 11‐year time‐points, complete data for the 12 CAPP predictors was available for 282 and 267 individuals, respectively. The model demonstrated good generalisability to predict asthma at both 8 and 11 years (AUC = 0.83 and 0.79, respectively) in the unselected MAAS subgroup (Table 1B; Figure 3). PPV also remained comparable in MAAS (PPV = 0.45 and 0.41, respectively), with further improvements reported in the high‐risk subgroup validations at both time‐points (Table 1B).

### 3.5 Sensitivity analysis

The CAPE and CAPP models were robust in correctly predicting non‐asthmatics using the alternative asthma definition (similar NPV). However, neither model was robust in predicting asthmatics, with an increase in false positive predictions reducing the PPV by approximately 50% for both models, likely due to disagreement between the original and modified asthma definitions (Table E6; Figure E1).

**FIGURE 4:**
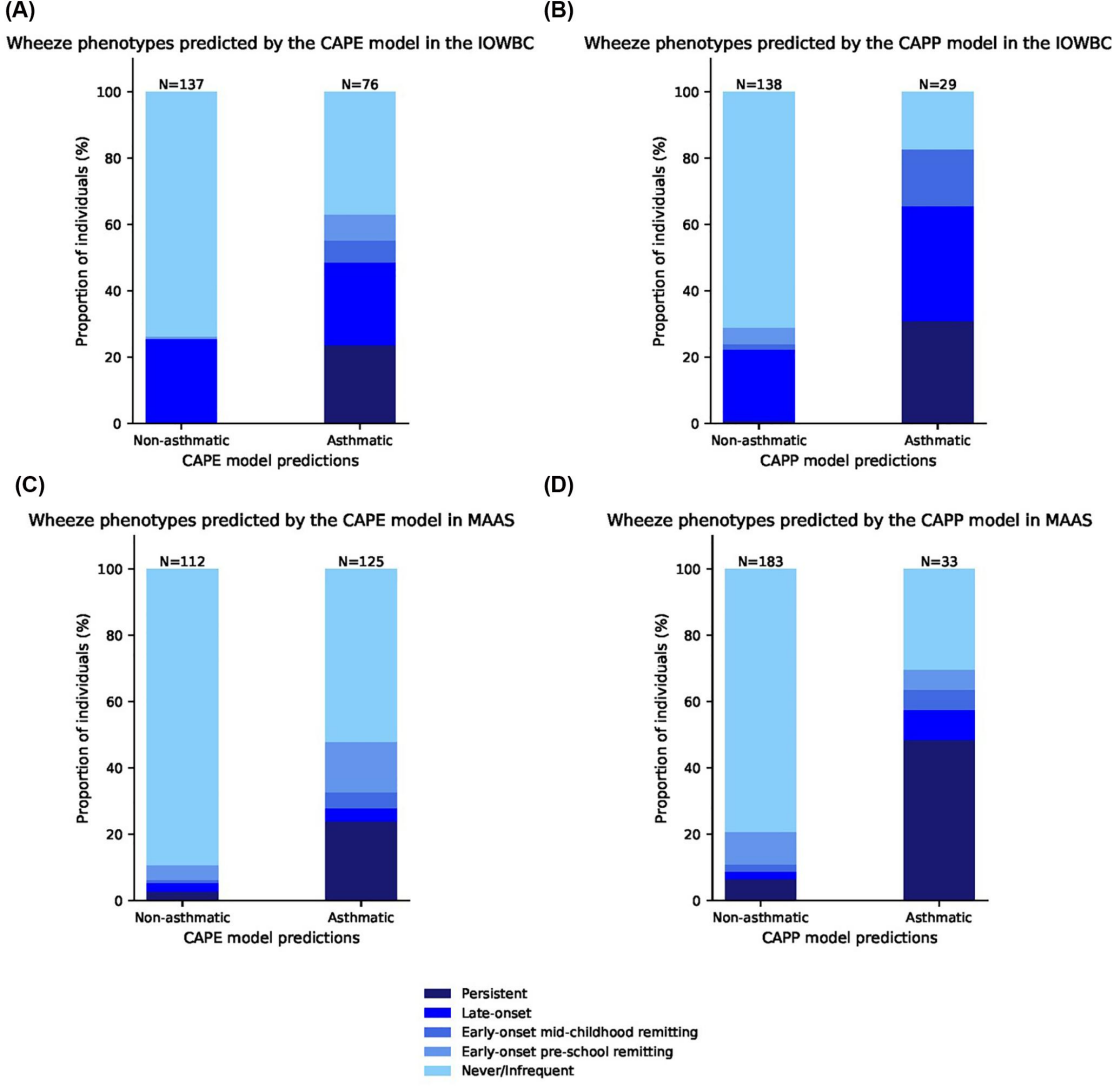
CAPE and CAPP model predictions and corresponding wheeze trajectories. The proportion of individuals corresponding to their most probable wheeze phenotype is presented for those predicted to be asthmatic or non‐asthmatic by the CAPE and CAPP models in the IOWBC (A, B) and MAAS (C, D)

Furthermore, both models showed excellent power to predict a persistent wheeze phenotype, with 100% and 90% of individuals with persistent wheeze offered a positive prediction by the CAPE and CAPP models in the IOWBC, respectively (90% and 57% in MAAS, respectively) (Figure 4).

### 3.6 Comparison with regression methods

Both the CAPE and CAPP models outperformed their equivalent logistic regression models (Table E7; Figure 3). There was a substantial decline in predictive performance of the CAPE‐logistic regression model (AUC = 0.59 vs. 0.71), with predictions being no better than chance in MAAS at 8 and 11 years (AUC = 0.47 and 0.49, respectively). Predictive power of the CAPP‐logistic regression model was also lower compared to the CAPP‐machine learning model (AUC = 0.76 vs. 0.82, PPV = 0.33 vs. 0.47).

Whilst the benchmark regression‐based model for the CAPE model (Persistent Asthma Predictive Score)^33^ was unable to be replicated due to lack of data on key predictors in the IOWBC, the model comparable with the CAPP model, PARS (Paediatric Asthma Risk Score),^34^ was replicated in the IOWBC and MAAS (AUC in IOWBC = 0.77, MAAS 8‐year = 0.79, MAAS 11‐year = 0.76). Among individuals with predictions available for both the CAPP and PARS models, positive net reclassification indices show that the proportion of reclassifications made by the CAPP model offered equal, if not greater, accuracy to predict future asthmatics than PARS in both the IOWBC (Table 2) and MAAS (Table E8).

**TABLE 2:** Reclassification table showing changes in prediction categorisation between the PARS and CAPP model

	Predicted risk (CAPP model)				Reclassified by CAPP (%)			
Predicted risk (PARS model)	No asthma	Asthma	Total		Increased risk	Decreased risk	Correctly reclassified	NRI
No asthma at age 10 (*n* = 149)								
No asthma	130	**9** ^b^	139					
Asthma	**1** ^a^	9	10		9 (6%)	1 (<1%)	1 (<1%)	−0.05
Total	131	18	149					
Asthma at age 10 (*n* = 25)								
No asthma	7	**8** ^a^	15					
Asthma	**0** ^b^	10	10		8 (32%)	0 (0%)	8 (32%)	0.32
Total	7	18	25					
				Total	17	1	9	

*Note*: Reclassification table comparing the change in individual asthma predictions with the CAPP model instead of the PARS model (reference model). For the PARS model, categorisations of predictions as asthmatic and non‐asthmatic was based on the optimal threshold (cutoff = 7) as defined in their original publication. Results are presented separately for individuals who were asthmatic and non‐asthmatic at age 10. Values in bold identify the number of individuals with disagreement in their asthma predictions made by the CAPP and PARS models. NRI = net reclassification index is given separately for true asthmatics and non‐asthmatics.

^a^ Reclassified into a more appropriate risk group by the CAPP model with respect to classifications made by the PARS model.

^b^ Reclassified into a less appropriate risk group by the CAPP model with respect to classifications made by the PARS model.

### 3.7 Explaining the “black‐box” models

Based on SHAP, only a subset of predictors included in each model were shown to have a major contribution on the predictions—early life cough and wheeze for the CAPE model and preschool cough, atopy and polysensitisation for the CAPP model (Figure E2). The contributions of these predictors were consistent with explanations of individual predictions (Figure E3). Redevelopment of the models including only these highly contributing predictors showed similar performance for the CAPP model but a 10% fall in AUC for the CAPE model (Figure E4).

## 4 DISCUSSION

### 4.1 Summary of findings

Two models, predicting school‐age asthma at age 10 within a general population, were developed using machine learning classification methods. The CAPE model uses a RBF SVM classifier and eight predictors to predict school‐age asthma in early life. The CAPP model uses a linear SVM classifier and twelve predictors available by age four. Both machine learning models offered superior predictive power and generalisability upon external validation compared to equivalent models developed using logistic regression methods as well as existing regression‐based models. Whilst the primary prediction outcome was school‐age asthma, both models demonstrated excellent sensitivity in predicting individuals likely to experience persistentwheeze throughout childhood.

### 4.2 Comparisons with existing models

To date, twenty‐one regression‐based prediction models have been developed for childhood asthma (reviewed in Kothalawala et al.^8^), of which only six have been externally validated (Table E9). A recent systematic review further identified 10 studies that developed prediction models for childhood asthma using machine learning approaches, but only eight specifically predicted school‐age asthma (5–14 years).^26^ Another study directly compared the performance of a current regression‐based asthma prediction model, PARS, with a conditional inference tree‐based decision rule model using the same predictors.^25^ However, none of these studies externally validated the machine learning models they proposed.

Similar to the CAPE and CAPP models, most published asthma prediction models are very good at ruling out asthma rather than ruling in asthma, resulting partly from low power due to low asthma prevalence.^8^ Even if existing models offer good PPV, this often degrades upon validation.^8^ Indeed, despite having similar asthma prevalence to existing studies in the original training set, the machine learning‐based CAPP model offered a 30% improvement in sensitivity compared to the only model suggested in asthma guidelines described to date (sensitivity: CAPP = 0.72 vs. loose Asthma Predictive Index = 0.42)^35^ and further 10% improvement in PPV compared to its benchmark model, PARS. This is consistent with Owora et al.‘s novel tree‐based model offering better predictive performance compared to an equivalent regression‐based PARS model (AUC = 0.85 vs. 0.71).^25^ Many of the other machine learning models also demonstrated greater performance to predict asthma than existing regression‐based models.^26^ However, with low sample sizes and indications of overfitting in many of these studies, the lack of external validation renders it impossible to evaluate any superior performance offered by these models, especially since they were all developed in high‐risk populations. Importantly, our CAPP machine learning model was more generalisable and retained its positive predictive power upon replication compared to its equivalent logistic regression model. Furthermore, reclassification tables comparing the CAPP and PARS models were suggestive of the CAPP model predicting future asthmatics more accurately than PARS, with a greater proportion of correct reclassifications than incorrect reclassification made by the CAPP model in both the IOWBC and MAAS. However, this needs to be confirmed within a larger cohort. The moderate but limited predictive power of the CAPE model compared to the CAPP model was unsurprising given the known difficulty of predicting the future development of childhood asthma using data from the first few years of life.^36^

### 4.3 Predictor selection and availability

Both the CAPE and CAPP models include data collected across multiple time‐points (Figures E5 and E6). Given the variable nature of asthma development and risk throughout early childhood, the consideration of predictors across multiple time‐points allowed for the identification of novel combinations of predictors that together improved the ability of the models to predict asthma. Whilst data collected across multiple time‐points may hinder the utility of the models, the selected predictors are all typically reported during routine health visits or tracked in child health records. Only the predictors of atopy and polysensitisation, which require a skin prick test (SPT), may restrict the applicability of the CAPP model in primary care. However, as these predictors are well‐established in the literature, were shown to make large contributions to the predictions (Figure E2), and resulted in a 10% reduction in AUC when excluded from the model (Table E10), the predictive benefit offered by the inclusion of sensitisation was deemed to outweigh the potential reduction in applicability.

Of the predictors selected for inclusion in the two models, some were well‐established risk factors with a clear inferred direction of asthma risk (Figure 2). Others were predictors which have not previously been used in childhood asthma prediction models (maternal age at the time of the child's birth, age of solid food introduction and total breastfeeding duration) and offer a less clear direction of asthma risk. The selection of these novel predictors, over others that have a more established biological relevance in the literature (such as parental asthma, eczema or allergic rhinitis), may be cautiously accepted by the clinical community. However, RFE identifies the subset of features that collectively offer the best predictive accuracy rather than devising a comprehensive list of childhood asthma risk factors, which may be biologically sound but lacking in predictive power.^37^ In fact, the predictors of wheeze and cough were among those repeatedly included in the majority of machine learning models identified to date.^26^ The predictors of atopy, polysensitisation and wheeze were also included in Owora et al.‘s machine learning model, however the predictors were taken from the PARS model rather than being identified from an independent feature selection.^25^ It is also important to acknowledge the possibility that the selection of these novel predictors may stem from an inherent bias of the random forest algorithm to assign greater importance to features which are continuous or which have a large number of categories.^38^ However, as the CAPE and CAPP models developed using these selected predictors demonstrated improved performance against existing prediction models, any bias stemming from the feature selection process did not appear to limit the inclusion of features that were truly predictive of school‐age asthma.

### 4.4 Prediction generalisability, robustness and resolution

In the unselected MAAS cohort, the CAPE and CAPP models showed moderate to good generalisability to predict asthma across school ages, despite the marginal decline in the PPV of the CAPE model. Validation in high‐risk MAAS subgroups showed the PPV of both models to increase with the number of allergic parents, suggesting that confidence in ruling in asthma improves in high‐risk groups; but replication in a larger study population is required.

The lack of a clear definition for asthma is an unavoidable limitation in epidemiological studies.^39^ The asthma definition used in this study aimed to account for children with a clinical indication of asthma (physician diagnosed) who were actively symptomatic, but also those potentially asymptomatic at the time of assessment due to the use of symptom relieving medications. Whilst both models were robust in predicting non‐asthmatics using an alternative asthma definition of wheeze and bronchial hyper‐responsiveness (BHR), they had reduced power to predict true asthmatics (∼50% decline in PPV). The latter may be explained by objective tests, such as spirometry and BHR, being influenced by treatment; potential asthmatics on controller medications, whom the models are trained to identify as asthmatic, may be considered as non‐asthmatic with the alternative definition, resulting in greater false positive predictions.

As the aim of this study was to compare whether machine learning approaches could improve upon existing regression‐based models that predict childhood asthma, the primary prediction outcome for this study was restricted to school‐age asthma rather than predicting asthma phenotypes. However, acknowledging the importance of exploring specific sub‐phenotypes of asthma, the resolution of the machine learning models to predict an individual's future wheeze trajectory was explored. Notably, both the CAPE and CAPP models showed excellent sensitivity to predict individuals with a persistent wheeze phenotype; these individuals would likely benefit from early primary or secondary asthma prevention/management.

To promote the clinical use of complex machine learning methods, studies must address the major hurdle of model interpretability. This study demonstrates how tools such as SHAP values^32^ can be used to unravel explanations of complex black‐box machine learning algorithms that have shown to improve the accuracy of childhood asthma predictions.

### 4.5 Strengths and limitations

This study had a number of strengths. First, each model was developed to make timely predictions to identify future asthmatics within a general population, rather than among those already considered at high‐risk (mainly those experiencing wheeze or with a familial history of asthma/allergy). Second, by utilising machine learning methods, novel predictor subsets for school‐age asthma were identified and the developed models offered improved predictive performance over current regression‐based methods. Third, to our knowledge, this is the first study to externally validate asthma prediction models developed using machine learning approaches. The models demonstrated good generalisability to predict school‐age asthma across multiple time‐points, without degrading the predictive power to rule in asthma (particularly with the CAPP model). Fourth, the two models displayed excellent sensitivity to predict a subgroup of individuals with persistent wheeze. Finally, this study was able to use SHAP to address one of the key issues preventing the uptake of machine learning methods in clinical practice—the inability to interpret the models and explain the individual predictions made.

However, this study was limited by both model development and validation being conducted in predominantly Caucasian populations. Machine learning also requires large datasets—the introduction of more data would undoubtedly improve the performance of the machine learning models and offer more precise performance estimates with smaller confidence intervals. To retain a sample size appropriate for machine learning, feature selection was conducted before performing a train‐test split. This decision could have resulted in information leakage, potentially biasing the performance seen in the IOWBC test sets. To mitigate any bias, external replication was used to evaluate the models; as performance in MAAS was similar to the IOWBC, data leakage was not deemed a significant problem. Finally, whilst genomic data was available in the IOWBC, only clinical and environmental predictors were considered in order to maximize the clinical applicability of the models. It is possible that the consideration of genomic predictors might significantly improve childhood asthma predictions further^22^ ^,^ ^40^; however, the aim of this study was to explore whether machine learning methods could surpass the predictive ceiling that existing logistic regression methods appeared to be limited to. Hence, to provide a fair comparison with existing regression‐based models, such asthma biomarkers were not incorporated into this study.

## 5 CONCLUSION AND FUTURE WORK

Using machine learning, the CAPE and CAPP models were able to surpass the predictive performance of similar models developed using traditional logistic regression‐based methods. Both models were generalisable in an independent population, with the CAPP model also demonstrating superior predictive power to rule in true asthmatics compared to its benchmark model (and was retained upon validation). Future application of these models could include the development of a personalised tool/app capable of providing explanations of which predictors contributed to an individual's predicted probability of developing asthma. Both models also demonstrated excellent sensitivity to predict a subgroup of persistent wheezers. Therefore, rather than developing an all‐encompassing asthma prediction tool, further research into predicting specific ‘asthmas’ using machine learning approaches may offer greater predictive insight and clinical utility. Finally, continued exploration of machine learning approaches and the identification and integration of novel biomarkers is warranted to further improve the power to predict future childhood asthma.

## CONFLICT OF INTEREST

The authors have no funding relationships or conflicts of interest related to this article to disclose.

## AUTHOR CONTRIBUTIONS

*Conceptualization and design*: Dilini M. Kothalawala, S. Hasan Arshad, Faisal I. Rezwan and John W. Holloway. *Data acquisition*: John W. Holloway, Angela Simpson, Adnan Custovic, S. Hasan Arshad and STELAR/UNICORN investigators. *Analysis and interpretation*: Dilini M. Kothalawala. *Manuscript draft*: Dilini M. Kothalawala. *Manuscript revision and final approval*: all authors.

## STELAR/UNICORN INVESTIGATORS

Graham C Roberts, Human Development and Health, Faculty of Medicine, University of Southampton, David Hide Asthma and Allergy Research Centre, Isle of Wight and NIHR Southampton Biomedical Research Centre, University Hospitals Southampton NHS Foundation Trust, Southampton, UK.

Steve W Turner, Child Health, University of Aberdeen, Aberdeen, UK.

Raquel Granell, MRC Integrative Epidemiology Unit, Population Health Sciences, Bristol Medical School, University of Bristol, UK.

Sadia Haider, National Heart and Lung Institute, Imperial College London, UK.

Sara Fontanella, National Heart and Lung Institute, Imperial College London, UK.

Paul Cullinan, National Heart and Lung Institute, Imperial College London, UK.

## Supporting information

Supporting Information S1

## References

[1] Akdis CA , Agache I . Global Atlas of Asthma. European Academy of Allergy and Clinical Immunology; 2013.

[2] Licari A , Castagnoli R , Brambilla I , et al. Asthma endotyping and biomarkers in childhood asthma. Pediatr Allergy Immunol Pulmonol. 2018;31:44‐55. 30069422 10.1089/ped.2018.0886 PMC6069590

[3] Pavord ID , Beasley R , Agusti A , et al. After asthma: redefining airways diseases. Lancet. 2018;391:350‐400. 28911920 10.1016/S0140-6736(17)30879-6

[4] Ullmann N , Mirra V , Di Marco A , et al. Asthma: differential diagnosis and comorbidities. Front Pediatr. 2018;6:276. 30338252 10.3389/fped.2018.00276 PMC6178921

[5] Isabella Annesi‐Maesano CS , Caillaud D , de Blay F , Lavaud F , Charpin D , Raherisson C . Factors related to under‐diagnosis and undertreatment of childhood asthma in metropolitan France. Multidiscip Respir Med. 2012;7. 10.1186/2049-6958-7-24 PMC3436684 22958936

[6] Bush A , Fleming L , Saglani S . Severe asthma in children. Respirology. 2017;22:886‐897. 28543931 10.1111/resp.13085

[7] Martinez FD , Taussig LM , Holberg CJ , Halonen M , Morgan WJ . Asthma and wheezing in the first six years of life. The Group Health Medical Associates. N Engl J Med. 1995;332:133‐138. 7800004 10.1056/NEJM199501193320301

[8] Kothalawala DM , Kadalayil L , Weiss VBN , et al. Prediction models for childhood asthma: a systematic review. Pediatr Allergy Immunol. 2020;31:616‐627. 32181536 10.1111/pai.13247

[9] James G , Witten D , Hastie T , Tibshiran R . An Introduction to Statistical Learning. 1st ed. Springer‐Verlag; 2013.

[10] Singal AG , Mukherjee A , Elmunzer BJ , et al. Machine learning algorithms outperform conventional regression models in predicting development of hepatocellular carcinoma. Am J Gastroenterol. 2013;108:1723‐1730. 24169273 10.1038/ajg.2013.332 PMC4610387

[11] Waljee AK , Higgins PD , Singal AG . A primer on predictive models. Clin Transl Gastroenterol. 2014;5:e44. 24384866 10.1038/ctg.2013.19 PMC3912317

[12] Prosperi MC , Marinho S , Simpson A , Custovic A , Buchan IE . Predicting phenotypes of asthma and eczema with machine learning. BMC Med Genomics. 2014;7(Suppl 1):S7. 25077568 10.1186/1755-8794-7-S1-S7 PMC4101570

[13] Finkelstein J , Jeong IC . Machine learning approaches to personalize early prediction of asthma exacerbations. Ann N Y Acad Sci. 2017;1387:153‐165. 27627195 10.1111/nyas.13218 PMC5266630

[14] Goto T , Camargo CA, Jr. , Faridi MK , Yun BJ , Hasegawa K . Machine learning approaches for predicting disposition of asthma and COPD exacerbations in the ED. Am J Emerg Med. 2018;36:1650‐1654. 29970272 10.1016/j.ajem.2018.06.062

[15] Patel SJ , Chamberlain DB , Chamberlain JM . A machine learning approach to predicting need for hospitalization for pediatric asthma exacerbation at the time of emergency department triage. Acad Emerg Med. 2018;25:1463‐70. 30382605 10.1111/acem.13655

[16] Saglani S , Custovic A . Childhood asthma: advances using machine learning and mechanistic studies. Am J Respir Crit Care Med. 2018;199:414‐422. 10.1164/rccm.201810-1956CI 30571146

[17] Chatzimichail EA , Rigas AG , Paraskakis EN . An artificial intelligence technique for the prediction of persistent asthma in children. In Proceedings of the 10th IEEE International Conference on Information Technology and Applications in Biomedicine; 2010:1‐4.

[18] Chatzimichail E , Paraskakis E , Rigas A . An evolutionary two‐objective genetic algorithm for asthma prediction. In 2013 UKSim 15th International Conference on Computer Modelling and Simulation; 2013:90‐94.

[19] Chatzimichail E , Paraskakis E , Sitzimi M , Rigas A . An intelligent system approach for asthma prediction in symptomatic preschool children. Computd Math Methods Med. 2013;2013:240182. 10.1155/2013/240182 PMC3612481 23573166

[20] Chatzimichail E , Paraskakis E , Rigas A . Predicting asthma outcome using partial least square regression and artificial neural networks. Adv Artif Intell. 2013:1‐7.

[21] Krautenbacher N , Flach N , Böck A , et al. A strategy for high‐dimensional multivariable analysis classifies childhood asthma phenotypes from genetic, immunological, and environmental factors. Allergy. 2019. 10.1111/all.13745 PMC6767756 30737985

[22] Fehrenbach H , Smolinska A , Klaassen EMM , et al. Profiling of volatile organic compounds in exhaled breath as a strategy to find early predictive signatures of asthma in children. PLoS One. 2014;9. 10.1371/journal.pone.0095668 PMC3994075 24752575

[23] AlSaad R , Malluhi Q , Janahi I , Boughorbel S . Interpreting patient‐specific risk prediction using contextual decomposition of BiLSTMs: application to children with asthma. BMC Med Inform Decis Mak. 2019;19:214. 31703676 10.1186/s12911-019-0951-4 PMC6842261

[24] Bose S , Kenyon CC , Masino AJ . Personalized prediction of early childhood asthma persistence: a machine learning approach. PLoS One. 2021;16:e0247784. 33647071 10.1371/journal.pone.0247784 PMC7920380

[25] Owora AH , Tepper RS , Ramsey CD , Becker AB . Decision tree‐based rules outperform risk scores for childhood asthma prognosis. Pediatr Allergy Immunol. 2021. 10.1111/pai.13530 33938038

[26] Patel D , Hall GL , Broadhurst D , Smith A , Schultz A , Foong RE . Does machine learning have a role in the prediction of asthma in children? Paediatr Respir Rev. 2021. 10.1016/j.prrv.2021.06.002 34210588

[27] Ahmad MA , Teredesai A , Eckert C . Interpretable machine learning in healthcare. In 2018 IEEE International Conference on Healthcare Informatics (ICHI); 2018:447.

[28] Arshad SH , Holloway JW , Karmaus W , et al. Cohort profile: the Isle of Wight Whole Population Birth Cohort (IOWBC). Int J Epidemiol. 2018;47:1043‐4i. 29547889 10.1093/ije/dyy023 PMC6124620

[29] Azur MJ , Stuart EA , Frangakis C , Leaf PJ . Multiple imputation by chained equations: what is it and how does it work? Int J Methods Psychiatr Res. 2011;20:40‐49. 21499542 10.1002/mpr.329 PMC3074241

[30] He H , Garcia EA , Adasyn SL . Adaptive synthetic sampling approach for imbalanced learning. In IEEE International Joint Conference on Neural Networks (IEEE World Congress on Computational Intelligence), Hong Kong; 2008:1322‐1328.

[31] Custovic A , Simpson BM , Murray CS , Lowe L , Woodcock A . The National Asthma Campaign Manchester Asthma and Allergy Study. Pediatr Allergy Immunol. 2002;13:32‐37. 12688622 10.1034/j.1399-3038.13.s.15.3.x

[32] Lundberg S , Lee S‐I . A Unified Approach to Interpreting Model Predictions. arXiv preprint. arXiv:170507874 2017.

[33] Vial Dupuy A , Amat F , Pereira B , Labbe A , Just J . A simple tool to identify infants at high risk of mild to severe childhood asthma: the Persistent Asthma Predictive Score. J Asthma. 2011;48:1015‐1021. 22022892 10.3109/02770903.2011.626481

[34] Biagini Myers JM , Schauberger E , He H , et al. A Pediatric Asthma Risk Score to better predict asthma development in young children. J Allergy Clin Immunol. 2018;143:1803‐1810e2. 30554722 10.1016/j.jaci.2018.09.037 PMC6504569

[35] Castro‐Rodríguez JAHC , Wright AL , Martinez FD . A clinical index to define risk of asthma in young children with recurrent wheezing. Am J Respir Crit Care Med. 2000;162:1403‐1406. 11029352 10.1164/ajrccm.162.4.9912111

[36] Global Initiative for Asthma . Global Strategy for Asthma Management and Prevention; 2018.

[37] Isabelle Guyon JW , Barnhill S . Gene selection for cancer classification using support vector machines. Mach Learn. 2002;46:389‐422.

[38] Strobl C , Boulesteix A‐L , Zeileis A , Hothorn T . Bias in random forest variable importance measures: illustrations, sources and a solution. BMC Bioinf. 2007;8. 10.1186/1471-2105-8-25 PMC1796903 17254353

[39] Van Wonderen KE , Van Der Mark LB , Mohrs J , Bindels PJ , Van Aalderen WM , Ter Riet G . Different definitions in childhood asthma: how dependable is the dependent variable? Eur Respir J. 2010;36:48‐56. 20032011 10.1183/09031936.00154409

[40] Klaassen EM , van de Kant KD , Jobsis Q , et al. Exhaled biomarkers and gene expression at preschool age improve asthma prediction at 6 years of age. Am J Respir Crit Care Med. 2015;191:201‐207. 25474185 10.1164/rccm.201408-1537OC

